# Stage-Specific Effects of Candidate Heterochronic Genes on Variation in Developmental Time along an Altitudinal Cline of *Drosophila melanogaster*

**DOI:** 10.1371/journal.pone.0011229

**Published:** 2010-06-18

**Authors:** Julián Mensch, Valeria Carreira, Nicolás Lavagnino, Julieta Goenaga, Guillermo Folguera, Esteban Hasson, Juan José Fanara

**Affiliations:** Laboratorio de Evolución, Departamento de Ecología, Genética y Evolución, Facultad de Ciencias Exactas y Naturales, Universidad de Buenos Aires, Buenos Aires, Argentina; University of Umeå, Sweden

## Abstract

**Background:**

Previously, we have shown there is clinal variation for egg-to-adult developmental time along geographic gradients in *Drosophila melanogaster*. Further, we also have identified mutations in genes involved in metabolic and neurogenic pathways that affect development time (heterochronic genes). However, we do not know whether these loci affect variation in developmental time in natural populations.

**Methodology/Principal Findings:**

Here, we constructed second chromosome substitution lines from natural populations of *Drosophila melanogaster* from an altitudinal cline, and measured egg-adult development time for each line. We found not only a large amount of genetic variation for developmental time, but also positive associations of the development time with thermal amplitude and altitude. We performed genetic complementation tests using substitution lines with the longest and shortest developmental times and heterochronic mutations. We identified segregating variation for neurogenic and metabolic genes that largely affected the duration of the larval stages but had no impact on the timing of metamorphosis.

**Conclusions/Significance:**

Altitudinal clinal variation in developmental time for natural chromosome substitution lines provides a unique opportunity to dissect the response of heterochronic genes to environmental gradients. Ontogenetic stage-specific variation in *invected*, *mastermind*, *cricklet* and *CG14591* may affect natural variation in development time and thermal evolution.

## Introduction

Understanding the ontogenetic trajectories of life-history traits is necessary to explain and predict constraints and variation in the evolution of characters. In this context, any variation in the state of individuals prior to reproduction will be preserved to some degree at the reproductive stage. In this sense, the time elapsed from the embryo to the reproductive phase, commonly known as developmental time (DT), is directly related to an individual's reproductive success [1].

In holometabolous insects like fruit flies, which occupy ephemeral habitats, the impact of DT on fitness is further exaggerated [2]. Although life history models assume that natural selection should favor the maximization of growth rate, genetic correlations with pre-adult viability [2], [3], adult body size [4], longevity [5] and starvation resistance [6] constraint the evolution of DT in the model organism *D. melanogaster*.

Changes in the timing of developmental processes – heterochrony – could account for many evolutionary changes among populations and species. However, the first criterion for heterochonic evolution is the existence of natural variation for developmental time. Several studies have shown latitudinal clinal variation for DT in *Drosophila melanogaster* populations of Australia [7] and South America [8], [9]. Also, Folguera *et al.* [8] reported that egg-to-adult DT was longer in southern localities and lowland populations, a pattern that leads to the hypothesis of adaptive thermal evolution.

DT is a quantitative trait determined by multiple segregating genes that are sensitive to temperature variation [10]. It has been also shown that heterochronic genes are mainly involved in metabolic and neurogenic pathways, such as *Hippo*, *Notch* and *insulin signaling pathway* [10], [11]. However, it is largely unknown whether these pathways affect variation in DT in natural populations. The resolution of this question may uncover the genetic architecture of this ecologically relevant trait and, hopefully, would provide insights into the evolutionary processes governing natural variation. Interestingly, *In(2L)t* and *In(2R)R*, two cosmopolitan polymorphic inversions of the second chromosome, affect DT [9], [12], and several mutants showing the strongest effects on DT mapped to the second chromosome [10]. Here, we assessed the effects of four genes mapping to the right arm of the second chromosome and that affect metabolism and neurogenesis on natural variation of DT. *invected* and *mastermind* are involved in the development of the nervous system [13], [14]. *cricklet* is a gene encoding a carboxylesterase involved in the metabolism of the juvenile hormone [15]. *CG14591* has no clear association with any biological process.

Genetic complementation testing is a potent tool to investigate the contribution of individual genes to natural variation in quantitative complex traits [16]. Furthermore, this experimental approach is particularly accurate when employing the chromosome substitution line technique, since natural chromosomes can be extracted from wild organisms and placed into a common genetic background. In this sense, *Drosophila* brings an impressive toolkit for dissecting multiple interacting loci with individually small effects that affect quantitative developmental traits [16].

We report the results of a survey of variation in DT using second chromosome substitution lines derived from sampling localities that lay along latitudinal and altitudinal gradients from several populations of *Drosophila melanogaster* of western Argentina. We show that variation in DT among populations is not only correlated with altitude but also with certain features of the thermal regime. Clinal variation in DT over short geographic distances and its association with climatic variables suggests that among-population differentiation is the consequence of different selective pressures along the altitudinal gradient. Also we show that allelic variants at candidate heterochronic genes *invected*, *mastermind*, *criclket* and *CG14591* contribute to altitudinal variation in DT with stage-specific effects.

## Materials and Methods

### Generating second chromosome substitution lines

**Figure 1:**
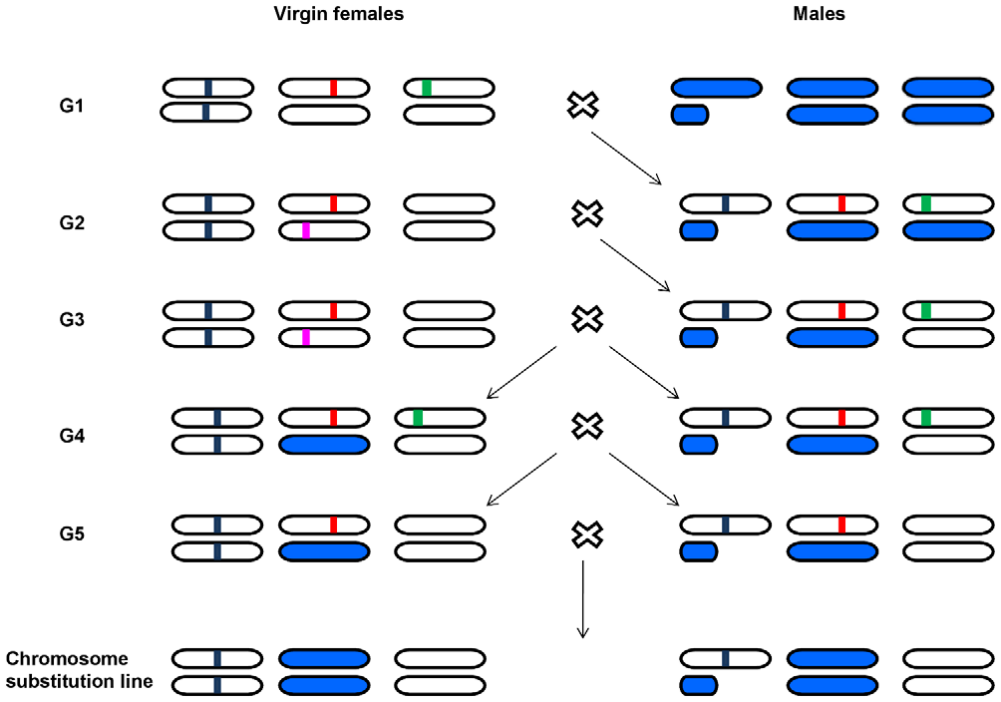
Crosses generating second chromosome substitution lines. *Canton S B* chromosomes are colored in white. Natural chromosomes are colored in blue. Small bars in chromosomes represent dominant phenotypic markers utilized in the isogenization of chromosomes: *Curly* (red), *Stubble* (green), *Sternopleural* (pink) and *white* eyes (blue). See Materials and Methods for more information.

Isofemale lines were founded by rearing the progeny of gravid *Drosophila melanogaster* females collected in six localities along latitudinal and altitudinal gradients in Western Argentina (February 2004 and February 2005). The geographical location, latitude, longitude, altitude and climatic information for each population are given in Table 1. All lines were maintained by full-sib mating for 10 generations on cornmeal-molasses-agar medium under standard conditions of 25±1°C, 70% humidity and a 12-h light∶ 12-h dark cycle. After 10 generations, a single second chromosome was extracted from each isofemale line and substituted into the genetic background of an isogenic *Canton-S B* strain by standard techniques using balancer chromosomes (Figure 1). To construct second chromosome substitution lines, one male of each isofemale line were crossed to *w*; *Cy*/Canton-S B; *Sb*/Canton-SB females [generation 1 (G1)]. A single *w*; *Cy*/+ 2; *Sb*/+ 3 male from the progeny of each cross was crossed to *w*; *Cy*/*Sp*; Canton-S B females. Next, *w*; *Cy*/+ 2; *Sb*/Canton-S B males were crossed to *w*; *Cy/Sp*; Canton-S B females (G3). Females and males of genotype *w*; *Cy*/+ 2; *Sb*/Canton-S B were intercrossed at G4, and the *Cy* and the *Sb* balancers were eliminated in the next generation (G5), obtaining an isogenic second chromosome substitution line with genotype *w*; +2; Canton S B. By means of this protocol we generated 50 second chromosome substitution lines isogenic for one wild derived chromosome in an otherwise isogenic background common to all lines. An average of 9 lines per population was obtained.

**Table 1:** Geographic and climatic information for each population studied.

Localities	Altitude	Latitude	Longitude	Minimal mean	Maximal mean	Mean	Thermal
	(meters)			temperature (°C)	temperature (°C)	temperature (°C)	amplitude (°C)
***Neuquén***	260	38°58′	68°08′	7.6	22.4	14.7	14.8
***Lavalle***	647	32°50′	68°28′	10.6	23.9	17	13.3
***Güemes***	739	24°38′	65°03′	10.6	24	16.6	13.4
***Chilecito***	1043	29°10′	67°28′	10.2	24.9	17.2	14.7
***San Blas***	1061	28°25′	67°06′	10.2	24.9	17.2	14.7
***Uspallata***	1915	32°35′	69°22′	2.7	21.2	11.6	18.5

### Developmental time assay

For each substitution line, 300 pairs of sexually mature flies were placed in egg collecting chambers for 8 hours. Eggs were allowed to hatch and batches of 30 first-instar larvae were transferred to culture vials containing 10 ml of cornmeal-agar-molasses medium (4 replicate vials per line). Emerged flies from each vial were collected every 12 hours and sorted by sex. We estimated DT as the time elapsed since the transfer of first-instar larvae to the vials until adult emergence. All vials were kept in an incubator at 25±1°C, under a 12-h light∶ 12-h dark cycle and at 70% humidity.

Forward stepwise multiple regression analyses were performed using geographic and climatic variables separately to test for clinal variation in developmental time. The geographic variables considered were altitude and latitude; and the climatic variables were minimal mean temperature, maximal mean temperature, mean temperature and thermal amplitude.

### Quantitative complementation tests

Quantitative complementation tests were performed to examine the contribution of *invected*, *cricklet*, *CG14591* and *mastermind* to natural variation in DT among the most divergent second chromosome substitution lines. Thus, from our set of isogenic lines we selected those exhibiting the most extreme phenotypes for DT (the fast and slow developing lines). These lines were crossed individually with lines carrying *P [GT1]* element mutated alleles in one of the four candidate genes identified by Mensch *et al.* (2008) [10] and, in parallel, with a *P [GT1]* element-free insertion line with the same genetic background (*Canton-S* B). The genotypes of the F1 progeny of the crosses were *m*/+_i_ and *Canton-S B*/+_i_, respectively, where *m* is a mutant allele in one of the candidate genes derived from a *P [GT1]* element insertion line and +_i_ represents a wild derived allele of the candidate gene. We used *P [GT1]* insertion lines BG00846, BG01339, BG01672 and BG01902 that carry mutant alleles for *invected*, *cricklet*, *CG14591* and *mastermind*, respectively [10].

We evaluated variation in developmental time among genotypes by means of a four-way analysis of variance (ANOVA) according to the mixed model:

Y = μ + L + S + G+ R (L ×S× G) + L × G + L × S + G × S + L × G × S + E,

where L, G and S are the fixed cross-classified effects of line (second chromosome substitution line), genotype (*Canton-S B* or *m*) and sex, respectively. R stands for the among replicate effect (random), and E is the error (within vial variance). The first criterion that must be met for quantitative failure of complementation is a significant Line by Genotype interaction. However, quantitative failure of complementation can be explained either as due to allelism or epistatic interactions [17], [18]. Thus, in order to identify allelic failure to complement, the second criterion to be met is that variation among lines in the *Canton-S* B background is not greater than variance among lines in the mutant background [17], [19]. If both criteria are met, it can be inferred that a candidate gene affects natural variation in developmental time.

In all complementation tests, DT was partitioned into larval developmental time (LDT) and pupal developmental time (PDT), involving the duration of all events that occurred before and after pupation, respectively. All statistical tests were performed using the STATISTICA package (StatSoft, Inc. 1999, 2001).

## Results

### Patterns of genetic variation among natural substitution lines

**Figure 2:**
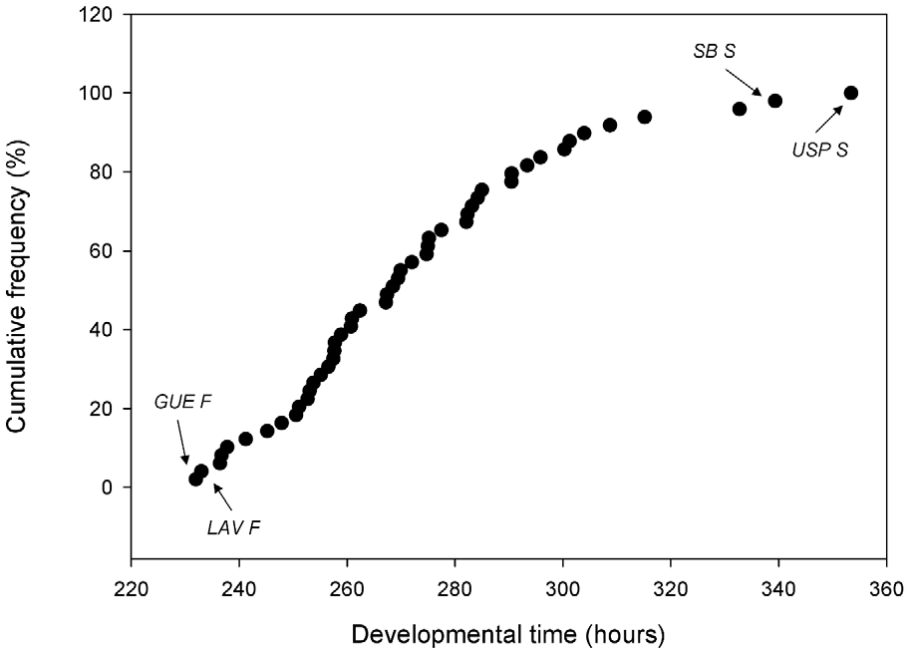
Variation in developmental time among second chromosome substitution lines. Distribution of genetic variation among the 50 second chromosome substitution lines. Arrows indicate the most divergent lines: *USP S* (*Uspallata* slow), *SB S* (*San Blas* slow), *LAV F* (*Lavalle* fast) and *GUE F* (*Güemes* fast).

A large amount of variation in DT was found among natural substitution lines, indicating that *D. melanogaster* second chromosome harbors genetic variation in natural populations for this fitness related trait (Figure 2). Among the lines showing the fastest phenotypes were *Güemes fast* (*GUE F*, 232 hours) and *Lavalle fast* (*LAV F*, 233 hours), whereas the lines that took more time to reach the adult stage were *San Blas slow* (*SB S*, 339 hours) and *Uspallata slow* (*USP S*, 353 hours).

**Figure 3:**
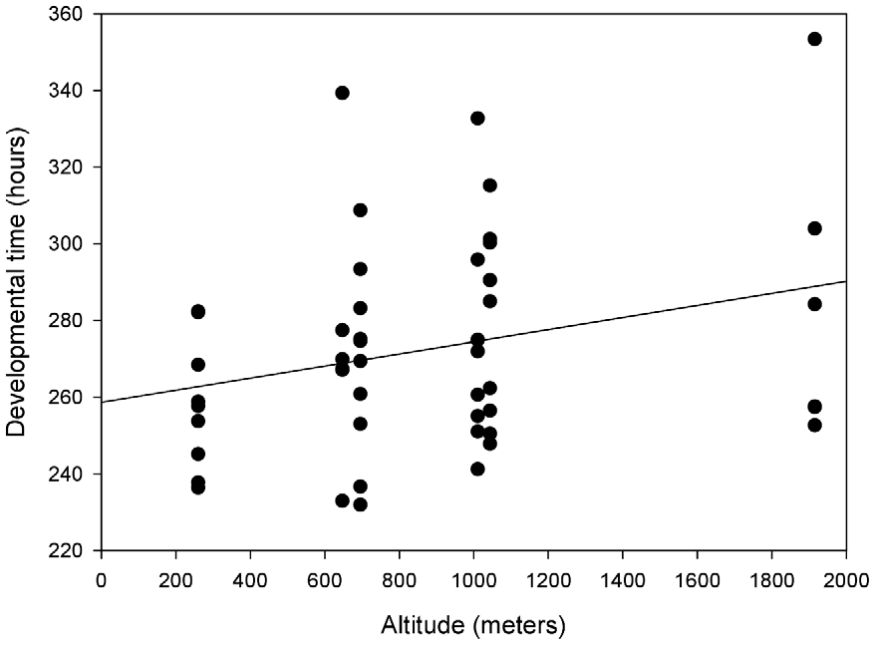
Regression of egg-to-adult developmental time on altitude. Positive association between egg-to-adult developmental time and altitude for the 50 second chromosome substitution lines studied.

**Figure 4:**
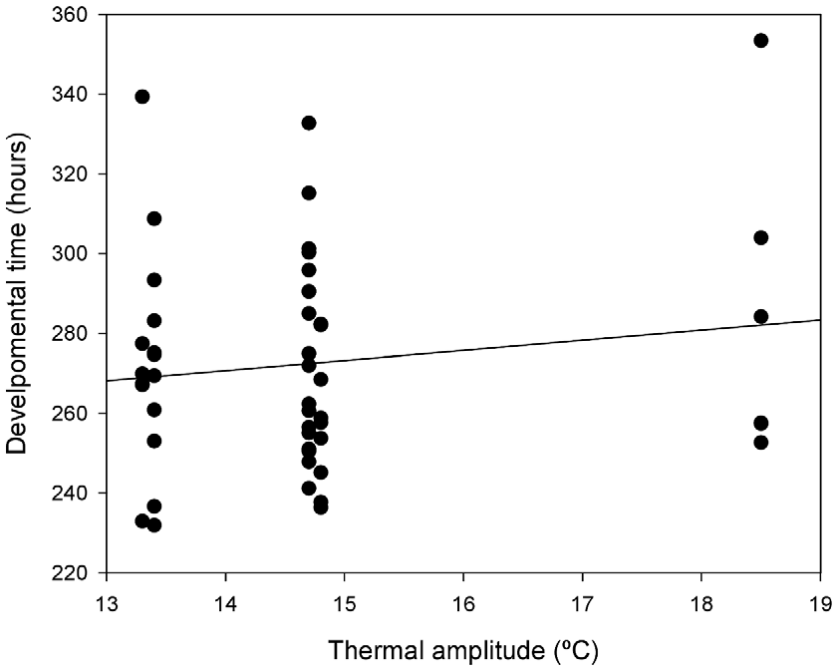
Regression of egg-to-adult developmental time on thermal amplitude. Positive association between egg-to-adult developmental time and thermal amplitude for the 50 second chromosome substitution lines studied.

Regression of DT on geographic variables revealed a significant and positive association between developmental time and altitude (r = 0.5, p<0.0001) (Figure 3) and none with latitude (p>0.05). Also, we detected a positive and significant association between DT and thermal amplitude (r = 0.26, p<0.0001) (Figure 4). In addition, altitude and thermal amplitude are positively correlated in the array of populations analyzed (r = 0.83, p<0.05) implying that greater daily thermal amplitude is likely to be found in highland populations. This pattern of genetic variation prompted us to explicitly test the hypothesis that altitude may be the main factor accounting for among population differentiation. Thus, we divided the complete set of populations sampled in two groups. The first group included sampling sites situated above 1000 meters above sea level: Uspallata, San Blas and Chilecito, which we to refer as highland populations from here on. The second group includes three lowland localities: Güemes, Lavalle and Neuquén which are located below 800 meters above sea level. Comparisons between these two groups showed that substitution lines from highland populations developed slower than lowland populations in males (*F*_1,185_ = 6.59; p = 0.01) and females (*F*_1,189_ = 7.72; p = 0.006). Indeed, DT substitution lines derived from highland and lowland sampling sites differed, on average, by 12 hours in both sexes.

In order to quantify within population genetic variation, we performed an ANOVA for each sampling locality (Table 2). In all populations, the Line factor was highly significant implying that the second chromosome harbors genetic variation for developmental time in all natural populations. Females developed significantly faster than males in Neuquén, Chilecito and Uspallata populations. The Line by Sex interaction term was not significant in any population.

**Table 2:** Analysis of variance for egg-to-adult developmental time for each population studied.

Population	Source of Variation	d.f.	*F*	*P*	σ^2^
***Güemes***	Line	9	9.41	0.001	63
	Sex	1	3.86	0.08	fixed
	Line × Sex	9	1.67	0.11	3
	Error	56			34
***Lavalle***	Line	5	44.97	<0.001	87
	Sex	1	4.49	0.08	fixed
	Line × Sex	5	1.36	0.26	1
	Error	36			12
***Neuquén***	Line	8	25.9	<0.0001	59
	Sex	1	31.2	<0.0001	fixed
	Line × Sex	8	0.42	0.89	0
	Error	50			41
***Chilecito***	Line	9	42.32	<0.0001	68
	Sex	1	16.7	<0.001	fixed
	Line × Sex	9	0.4	0.92	0
	Error	57			32
***Uspallata***	Line	5	60.7	<0.0001	77
	Sex	1	18.1	0.007	fixed
	Line × Sex	5	0.4	0.81	0
	Error	35			23
***San Blas***	Line	7	28.6	<0.0001	80
	Sex	1	2.43	0.16	fixed
	Line × Sex	7	1.16	0.33	0
	Error	47			20

**σ^2^: component of variance; d.f.: degree of freedom.**

### Candidate genes for altitudinal variation in developmental time

Since we detected great genetic differentiation between highland and lowland populations, we decided to test whether variation in candidate genes previously identified [10] could account, at least partially, for the clinal pattern. To this end we selected substitution lines with the most divergent DT for four independent quantitative complementation tests (QCTs) employing lines bearing heterochronic mutant alleles in a set of four genes, *mastermind*, *invected*, *cricklet* and *CG14591*, that mapped in the second chromosome. Thus, natural genotypes that differed in DT by more than five days were used in four independent QCTs. Our results showed a significant line by genotype interaction in each QCT suggesting a failure of complementation in all four candidate genes (Table 3). Likewise, in each analyses, L × G × S terms were not significant (Table 3) implying that genetic variation affects both sexes in similar way. In addition, in each QCT, variance among wild chromosomes was similar between mutant and *Canton-S B* backgrounds (Table 4), meaning that failure to complementation can mainly be attributed to allelism, rather than epistasis, as the genetic mechanism generating the phenotypic differences among lines.

**Table 3:** Analysis of variance of quantitative complementation tests for egg-to-adult developmental time.

*invected*				*mastermind*			
Source	d.f.	*F*	*P*	Source	d.f.	*F*	*P*
Line	3	4	0.012	Line	3	23.22	<0.00001
Genotype	1	68.95	<0.00001	Genotype	1	78.72	<0.00001
Line × Genotype	3	25.54	<0.00001	Line × Genotype	3	17.89	<0.00001
Rep(L × G × S)	48	3.64	<0.00001	Rep(L × G × S)	47	3.74	<0.00001
Sex	1	1.99	0.164	Sex	1	1.6	0.21
Line × Sex	3	1.37	0.261	Line × Sex	3	0.87	0.46
Genotype × Sex	1	0	1	Genotype × Sex	1	0.05	0.83
Line × Gen × Sex	3	0.5	0.684	Line × Gen × Sex	3	0.86	0.47
Error	739			Error	740		

*cricklet*				*CG14591*			
Source	d.f.	*F*	*P*	Source	d.f.	*F*	*P*
Line	3	28.08	<0.00001	Line	3	8.31	<0.00001
Genotype	1	87.57	<0.00001	Genotype	1	0.53	0.47
Line × Genotype	3	9.58	<0.00001	Line × Genotype	3	27.55	<0.00001
Rep(L × G × S)	48	4.66	<0.00001	Rep(L × G × S)	45	7.72	<0.00001
Sex	1	0.4	0.53	Sex	1	1.05	0.31
Line × Sex	1	0.58	0.45	Line × Sex	1	0.5	1
Genotype × Sex	3	0.84	0.96	Genotype × Sex	3	0	0.69
Line × Gen × Sex	3	0.1	0.49	Line × Gen × Sex	3	0.32	0.81
Error	760			Error	754		

**Table 4:** *F* statistics testing variances over mutant and *Canton S B* backgrounds.

Gene	Ratio	d.f.	*F*_DT_	*F*_LDT_	*F*_PDT_	critical *F*
*invected*	σ^2^ *_Canton S_*_/+i_/σ^2^ *_m_*_/+i_	3.3	2.48	0.89	0.91	9.28
*mastermind*	σ^2^ *_Canton S_*_/+i_/σ^2^ *_m_*_/+i_	3.3	2.11	0.88	0.94	9.28
*cricklet*	σ^2^ *_Canton S_*_/+i_/σ^2^ *_m_*_/+i_	3.3	1.17	0.87	0.92	9.28
*CG14591*	σ^2^ *_Canton S_*_/+i_/σ^2^ *_m_*_/+i_	3.3	0.54	0.99	0.94	9.28

All *F* values are below the critical *F*, meaning that variances over mutant and *Canton S B* backgrounds are similar for all candidate genes.

**Figure 5:**
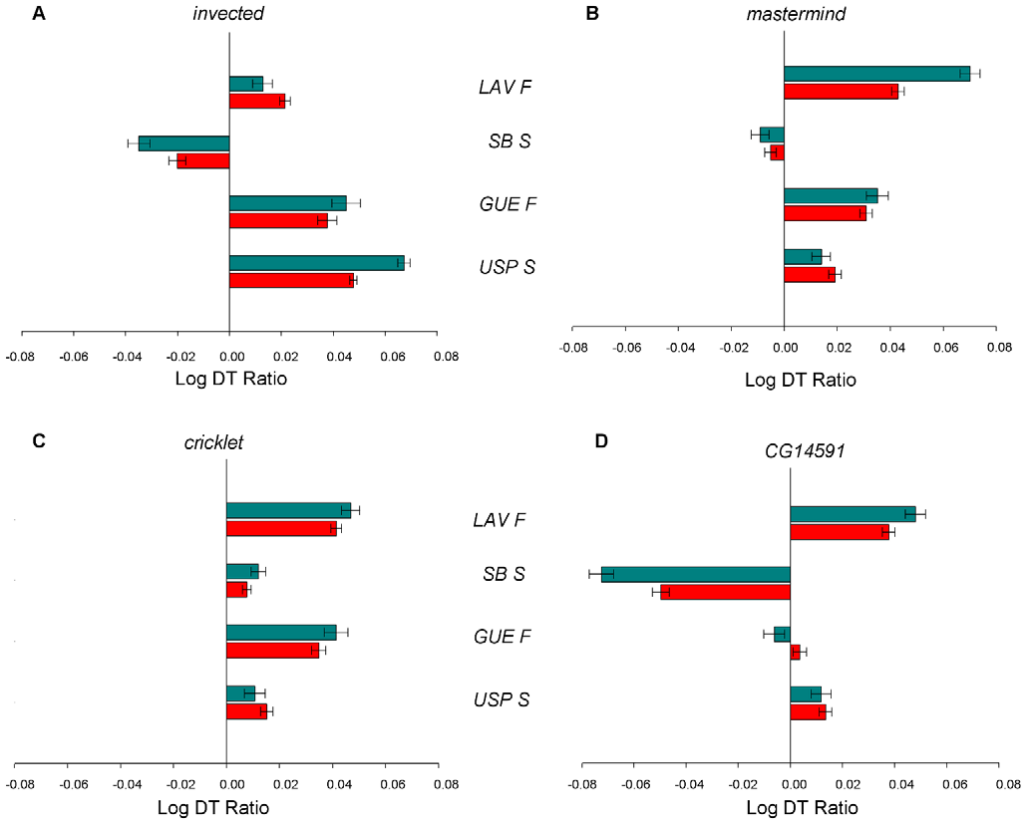
Quantitative complementation tests. Developmental time ratios for each line over a heterochronic mutant allele relative to that line over a *Canton S B* allele. The heterogeneity in the magnitude and direction of the DT ratios represents the genetic variation for each heterochronic gene. A) *invected*. B) *mastermind*. C) *cricklet*. D) *CG1491*. Blue bars represent larval developmental time and red bars refer to egg-to-adult developmental time.

Genetic variation in candidate genes was revealed by the different responses of natural chromosomes when combined with mutant alleles or the functional *Canton-S B* allele. Effects on DT of different wild chromosomes over mutant and *Canton SB* backgrounds are expressed as ratios for each QCT (Figure 5). Most ratios showed positive values indicating that wild chromosomes had longer DT on a mutant background than on *Canton-S B*, the only exception being line *SB S* in the QCTs for *invected*, *mastermind* and *CG14591*. Nevertheless, in the QCT for *CG14591*, we also identified a line (*GÜE F*) with a DT ratio close to zero, suggesting that this chromosome did not contribute to natural genetic variation for this gene. All in all, our results not only confirm that *mastermind*, *invected*, *cricklet* and *CG14591* are quantitative candidate genes for developmental time expression, but also that these genes exhibit natural allelic variants that contribute to natural variation in DT.

### Stage-specific variation in developmental time

In all QCTs, we further dissected egg-to-adult developmental time by measuring the duration of the larval (LDT) and pupal (PDT) stages. For all candidate genes, we found that the L × G interaction was significant for LDT but not for PDT (Table 5 and Table 6, respectively), indicating that segregating genetic variation mostly affected the time spent in premetamorphic stages rather than the duration of metamorphosis itself. As it can be observed in Figure 5, LDT accounts for the largest proportion of egg-to-adult DT genetic variation in these lines (blue and red bars, respectively). Once again, variance among wild chromosomes was similar between mutant and *Canton-S B* backgrounds (Table 4), supporting the idea that allelism is the best explanation for the genetic variation observed rather than epistasis.

**Table 5:** Analysis of variance of quantitative complementation tests for larval developmental time.

*invected*				*mastermind*			
Source	d.f.	*F*	*P*	Source	d.f.	*F*	*P*
Line	3	18.95	<0.00001	Line	3	63.06	<0.00001
Genotype	1	81.32	<0.00001	Genotype	1	144.61	<0.00001
Line × Genotype	3	76.5	<0.00001	Line × Genotype	3	48.39	<0.00001
Rep(L × G)	24	4.23	<0.00001	Rep(L × G)	24	4.99	<0.00001
Error	908			Error	908		

*cricklet*				*CG14591*			
Source	d.f.	*F*	*P*	Source	d.f.	*F*	*P*
Line	3	70.2	<0.00001	Line	3	29.6	<0.00001
Genotype	1	142.17	<0.00001	Genotype	1	0	NS
Line × Genotype	3	13.18	<0.00001	Line × Genotype	3	90.72	<0.00001
Rep(L × G)	24	4.81	<0.00001	Rep(L × G)	23	6.69	<0.00001
Error	916			Error	905		

**Table 6:** Analysis of variance of quantitative complementation tests for pupal developmental time.

*invected*				*mastermind*			
Source	d.f.	*F*	*P*	Source	d.f.	*F*	*P*
Line	3	3*10−3	NS	Line	3	2*10−4	NS
Genotype	1	1*10−2	NS	Genotype	1	4*10−3	NS
Line × Genotype	3	1*10−3	NS	Line × Genotype	3	7*10−4	NS
Error	25			Error	25		

*cricklet*				*CG14591*			
Source	d.f.	*F*	*P*	Source	d.f.	*F*	*P*
Line	3	1*10−3	NS	Line	3	4*10−2	NS
Genotype	1	1*10−2	NS	Genotype	1	2*10−2	NS
Line × Genotype	3	1*10−3	NS	Line × Genotype	3	5*10−2	NS
Error	25			Error	24		

## Discussion

### Variation in DT in second chromosomes and candidate genes

Developmental time is a fundamental life history trait closely linked to fitness, a short juvenile period is beneficial since it reduces the risks of predation and/or infections and has the advantage of earlier reproduction [1]. Life history models assume that natural selection should favor the maximization of growth rate. However, genetic variation within and between populations has been frequently identified for developmental time, suggesting that DT in a particular environment is not necessarily the maximum and that rapid growth rates may have an associated fitness cost.

Our survey of DT variation in substitution lines bearing different wild second chromosomes, in an otherwise identical genetic background, revealed a considerable amount of variation. The lines exhibiting the most extreme scores for DT differed by as much as 5 days. Moreover, our survey shows that chromosome *2* harbors an important portion of natural genetic variation affecting DT in all populations analyzed. In fact, the contribution of differences among chromosome *2* substitution lines to egg-to-adult DT variation ranged from 59% to 87% of the total phenotypic variance in the set of populations studied (Table 2).

Also interesting is that the line by sex interaction did not contribute to variation in DT in any of the localities, suggesting the lack of genetic variation for sexual dimorphism of DT. Interestingly, Mensch *et al.* (2008) [10] demonstrated a null input of the genotype by sex interaction to total phenotypic variance using a set of lab lines, as did the study of Van't Land *et al.*(1999) [9] showing clinal variation in South American populations. The absence of genetic variation for sexual dimorphism in developmental time has often been observed in other *drosophilids* as well [20], [21].

Regression analyses of DT on geographic and climatic variables indicated that altitudinal clines and the association with thermal amplitude reported in Folguera *et al.* (2008) [8] could be partially accounted for by allelic variants in genes located in the second chromosome. The associations between DT and altitude and thermal amplitude are congruent since daily mean thermal amplitude is likely to be more common in highland than in lowland sampling sites [8]. Further analysis comparing highland (after grouping localities situated above 1000 meters above sea level) and lowland (below 1000 meters above sea level) populations showed that DT in highland substitution lines was, on average, 12 hours longer.

**Figure 6:**
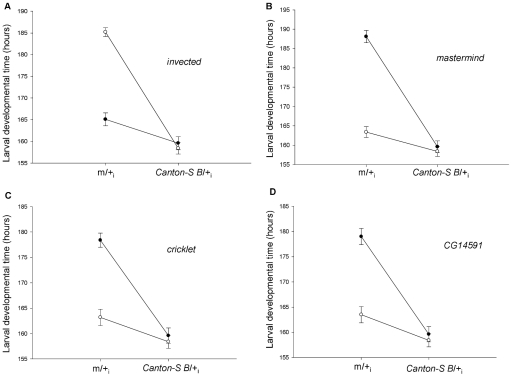
Contrasting pattern of altitudinal genetic variation among heterochronic genes. Black circles represent *Lavalle fast* alleles and white circles refer to *Uspallata slow* alleles. m refers to a mutated allele of a candidate gene. A) *invected*/*Uspallata slow* genotype exhibited a longer larval developmental time than the rest of the genotypes analyzed in the quantitative complementation test, indicative of genetic variation for this gene. B–D) In contrast, *m*/*Lavalle fast* (*mastermind*, *cricklet* and *CG14591*) genotypes showed a longer larval developmental time than the rest of the genotypes in the other quantitative complementation tests.

One of the major challenges to understand the genetic basis of complex adaptive traits (i.e. developmental time) has been the difficulty in mapping individual genes affecting natural genetic variation because differences in genetic background can profoundly affect complex phenotypes [22], [23]. Nevertheless, some successful efforts [24]–[27] allowed the identification of candidate genes for diverse traits. It is important to note that most of these studies used chromosomes carrying deficiencies [22] that were induced in heterogeneous collections of genetic backgrounds. In contrast, our study was performed with chromosome substitution lines that share the same genetic background either among them and with *P* element insertion lines that we used to characterize heterochronic candidate genes [10]. Failure of complementation in QCTs revealed that *invected*, *mastermind*, *cricklet* and *CG14591* have natural allelic variants that affect DT. To our knowledge this the first time that natural allelic variation is described for these genes as well as the (partial) characterization of the genetic basis of DT variation in natural populations. Moreover, the similar variances of natural allelic variants when combined with mutant and *Canton S B* alleles, in all QCTs, suggest an additive effect of allelic variants of *mastermind*, *cricklet*, *CG14591* and *invected* on DT. However, non additive effects due to interactions with other second chromosome loci affecting DT cannot be ruled out. Notice, for instance, that the genotype Lavalle/*mutant*, exhibited faster larval developmental time than the *Uspallata*/*mutant* genotype, when the mutant allele is contributed by the *P-element* insertion line for *invected* (Figure 6). On the other hand, the *Lavalle* chromosome prolonged and *Uspallata* chromosome speeded up development when combined in genotypes with mutant alleles contributed by the *P-element* insertion lines for *mastermind*, *cricklet* and *CG14591* (Figure 6). These results suggest that complex genetic interactions among several developmental pathways are affecting DT.

Noteworthy, two of the genes, *invected* and *mastermind* are expressed early in development and also during the larval and pupal stages [14], [28]. The former, a paralog of the pair-rule gene *engrailed*, is a transcription factor that is expressed in neuroectodermic cells [13], while *mastermind* is a key component of the *Notch* signaling pathway, which determines cell fate and regulates pattern formation. Likewise, *cricklet* (*CG9858*, also known as *EstR*) encodes a carboxylesterase that has been proposed to be associated with Juvenile hormone (JH) functions [15]. Yet, recent studies have shown poor kinetics towards various forms of JH [29]. Finally, no clear association with any biological process has been reported for *CG14591*. Our study provides, therefore, the first record concerning functional significance of *CG14591* in *D. melanogaster*.

### Holometabolous ontogeny and the nature of variation in DT

As a complex trait, DT displays considerable genetic correlations with other life history and morphological characters. Particularly, it has been suggested that DT may be involved in a trade-off with body size, since attaining a large size may imply a longer feeding period, and thus a longer DT. Thus, variation in growth patterns, growth rate and the duration of the growth period, affect the age and size at maturity, implying that the study of juvenile growth may be crucial to understand life history evolution [30]. In holometabolous insects, body size and development time are controlled by three factors: i) growth rate, ii) the time required to attain a critical size (related to the cessation of secretion of Juvenile hormone) and iii) the interval to cessation of growth (related to the timing of ecdysteroid secretion) which corresponds to the onset of pupation and metamorphosis [31]. Early in the last larval stage, larvae reach a critical size (at which point they have acquired enough nutrients to complete development), stop feeding and search for a pupation site. However, the cessation of feeding does not occur immediately, and there is a delay between the attainment of critical size and pupation. Thus, total developmental time can be conceived as the sum of the duration of successive discrete phases: embryonic, larval and pupal stages that end with the emergence of the imago. Our results illustrate that allelic variation in the four candidate genes studied only affected the time interval between the egg stage and pupation, which we call Larval Developmental Time (LDT) but not the interval between the pupal stage to adult emergence, Pupal Developmental Time (PDT). This contrasting pattern of variation along the ontogeny provides an initial insight to understand the genetic architecture underlying developmental time. In this sense, our results concur with the idea that variation in LDT may no have direct effect on the duration of the pupal stage [32]. Instead, since resource acquisition and growth are confined to the larval stages, LDT variation might directly affect adult body size [33]. This might be the case of *invected* alleles that not only affected larval developmental time but also influenced various adult body size traits [34].

The recognition that a single trait, DT, may be subdivided in “subtraits” that are not under the control of the same genes imply that the duration of larval and pupal stages responded to different selective pressures; a matter that may have broad implications in evolutionary genetics, since responses to selection may crucially depend on the genetic variance/covariance of individual “sub-traits” [32]. In this context, thermal selection is usually invoked as a causal factor for the frequently reported geographic clines of body size in *Drosophila* [7], [8], [11] however whether thermal selective pressure exerts a direct influence on LDT (reversed Bergman rule) or, actually, the target of selection is adult body size itself (Bergman rule) remains unclear. In this sense, a recent study conducted in our lab has shown that LDT and PDT respond differentially to thermal variation (Folguera *et al.*, unpublished results). Noteworthy, *mastermind*, *cricklet* and *CG14591* were also shown to be candidates for the plastic responses of DT to temperature variation [10], meaning that the effects of each gene depend on ambient temperature along ontogeny. All in all, thermal selection along the altitudinal cline may influence the large genetic variation in metabolic and neurogenic genes that affect LDT. In summary, our results are consistent with the hypothesis that egg-to-adult development time should not be considered as the sum of additive “sub-traits”. Indeed, as development is a complex time-dependent process, a system genetics approach would be appropriate in order to test the hypothesis of stage-specific developmental time variation at the genomic level. To this end, our study provides particular candidate genetic pathways to be tested in future studies taking advantage of the abundant genomic information available in *D. melanogaster* and allied species [35], [36].

## References

[1] Stearns SC (1992). The evolution of life histories.

[2] Chippindale AK, Alipaz JA, Chen H, Rose M (1997). Experimental evolution of accelerated development in *Drosophila*. 1. Developmental speed and larval survival.. Evolution.

[3] Prasad NG, Shakarad M, Anitha D, Rajamani M, Joshi A (2001). Correlated responses to selection for faster development and early reproduction in *Drosophila*: the evolution of larval traits.. Evolution.

[4] Nunney L (1996). The response to selection for fast larval development in Drosophila melanogaster and its effect on adult weight: an example of a fitness trade-off.. Evolution.

[5] Chippindale AK, Alipaz JA, Rose MR, Rose MR, Passananti HB, Matos M (2004). Experimental evolution of accelerated development in *Drosophila*. 2. Adult fitness and the fast development syndrome.. Methuselah flies: A case study in the evolution of aging.

[6] Harshman LG, Hoffmann AA, Clark AG (1999). Selection for starvation resistance in *Drosophila melanogaster*: physiological correlates, enzyme activities and multiple stress responses.. J Evol Biol.

[7] James AC, Partridge L (1995). Thermal evolution of the rate of larval development in *Drosophila melanogaster* in laboratory and fields populations.. J Evol Biol.

[8] Folguera G, Ceballos S, Spezzi L, Fanara JJ, Hasson E (2008). Clinal variation in developmental time and viability, and the response to thermal treatments in two species of *Drosophila*.. Biol J Linn Soc.

[9] Van 't Land J, Van Putten P, Zwaan B, Kamping A, Van Delden W (1999). Latitudinal variation in wild populations of *Drosophila melanogaster*: heritabilities and reaction norms.. J Evol Biol.

[10] Mensch J, Lavagnino N, Carreira VP, Massaldi A, Hasson E (2008). Identifying candidate genes affecting developmental time in *Drosophila melanogaster*: pervasive pleiotropy and gene-by-environment interaction.. BMC Dev Biol.

[11] De Jong G, Bochdanovits Z (2003). Latitudinal clines in *Drosophila melanogaster*: body size, allozyme frequencies, inversion frequencies, and the insulin-signalling pathway.. J Genet.

[12] Van Delden W, Kamping A (1991). Changes in relative fitness with temperature among second chromosome arrangements *in Drosophila melanogaster*.. Genetics.

[13] Gustavson E, Goldsborough AS, Ali Z, Kornberg TB (1996). The *Drosophila* engrailed and invected genes: partners in regulation, expression and function.. Genetics.

[14] Bettler D, Pearson S, Yedvobnick B (1996). The nuclear protein encoded by the *Drosophila* neurogenic gene mastermind is widely expressed and associates with specific chromosomal regions.. Genetics.

[15] Campbell PM, Harcourt RL, Crone EJ, Claudianos C, Hammock BD (2001). Identification of a juvenile hormone esterase gene by matching its peptide mass fingerprint with a sequence from the *Drosophila* genome project.. Insect Biochem Mol Biol.

[16] Mackay TF (2004). Complementing complexity.. Nat Genet.

[17] Paaby AB, Schmidt PS (2008). Functional significance of allelic variation at methuselah, an aging gene in *Drosophila*.. PLoS One.

[18] Edwards AC, Mackay TF (2009). Quantitative trait loci for aggressive behavior in *Drosophila melanogaster*.. Genetics.

[19] Geiger-Thornsberry GL, Mackay TF (2004). Quantitative trait loci affecting natural variation in *Drosophila* longevity.. Mech Ageing Dev.

[20] Soto EM, Soto IM, Carreira VP, Fanara JJ, Hasson E (2008). Host-related life history traits in interspecific hybrids of cactophilic *Drosophila*.. Entomologia Experimentalis et Applicata.

[21] Fanara JJ, Folguera G, Iriarte PF, Mensch J, Hasson E (2006). Genotype by environment interactions in viability and developmental time in populations of cactophilic *Drosophila*.. J Evol Biol.

[22] Mackay TF (2001). Quantitative trait loci in *Drosophila*.. Nat Rev Genet.

[23] Anholt RR, Mackay TF (2004). Quantitative genetic analyses of complex behaviours in *Drosophila*.. Nat Rev Genet.

[24] Fanara JJ, Robinson KO, Rollmann SM, Anholt RR, Mackay TF (2002). Vanaso is a candidate quantitative trait gene for *Drosophila* olfactory behaviour.. Genetics.

[25] Harbison ST, Chang S, Kamdar KP, Mackay TF (2005). Quantitative genomics of starvation stress resistance in *Drosophila*.. Genome Biol.

[26] Jordan KW, Carbone MA, Yamamoto A, Morgan TJ, Mackay TF (2007). Quantitative genomics of locomotor behavior in *Drosophila melanogaster*.. Genome Biol.

[27] Wang P, Lyman RF, Shabalina SA, Mackay TF, Anholt RR (2007). Association of polymorphisms in odorant-binding protein genes with variation in olfactory response to benzaldehyde in *Drosophila*.. Genetics.

[28] Schmid AT, Tinley TL, Yedvobnick B (1996). Transcription of the neurogenic gene mastermind during *Drosophila* development.. J Exp Zool.

[29] Crone EJ, Sutherland TD, Campbell PM, Coppin CW, Russell RJ (2007). Only one esterase of *Drosophila melanogaster* is likely to degrade juvenile hormone in vivo.. Insect Biochem Mol Biol.

[30] Gotthard K, Atkinson D, Thorndyke M (2001). Growth strategies of ectothermic animals in temperate environments.. Environmental and animal development.

[31] Davidowitz G, Nijhout HF (2004). The physiological basis of reaction norms: the interaction between growth rate, the duration of growth and body size.. Integrative and Comparative Biology.

[32] Nunney L (2007). Pupal period and adult size in *Drosophila melanogaster*: a cautionary tale of contrasting correlations between two sexually dimorphic traits.. J Evol Biol.

[33] Edgar B (2006). How flies get their size: genetics meets physiology.. Nat Rev Genet.

[34] Carreira V, Mensch J, Fanara JJ (2009). Body size in *Drosophila*: genetic architecture, allometries and sexual dimorphism.. Heredity.

[35] Mackay TF, Richards S, Gibbs R (2008). Proposal to sequence a *Drosophila* genetic reference panel: A community resource for the study of genotypic and phenotypic variation.. http://flybase.org/static_pages/news/whitepapers/Drosophila_Genetic_Reference_Panel_Whitepaper.pdf.

[36] *Drosophila* 12 Genomes Consortium (2007). Evolution of genes and genomes on the *Drosophila* phylogeny.. Nature.

